# Supplementary material for: A patatin-like phospholipase mediates Rickettsia parkeri escape from host membranes
Source: Nat Commun. 2022 Jun 27;13:3656. doi: 10.1038/s41467-022-31351-y (PMC9237051; doi:10.1038/s41467-022-31351-y)

a

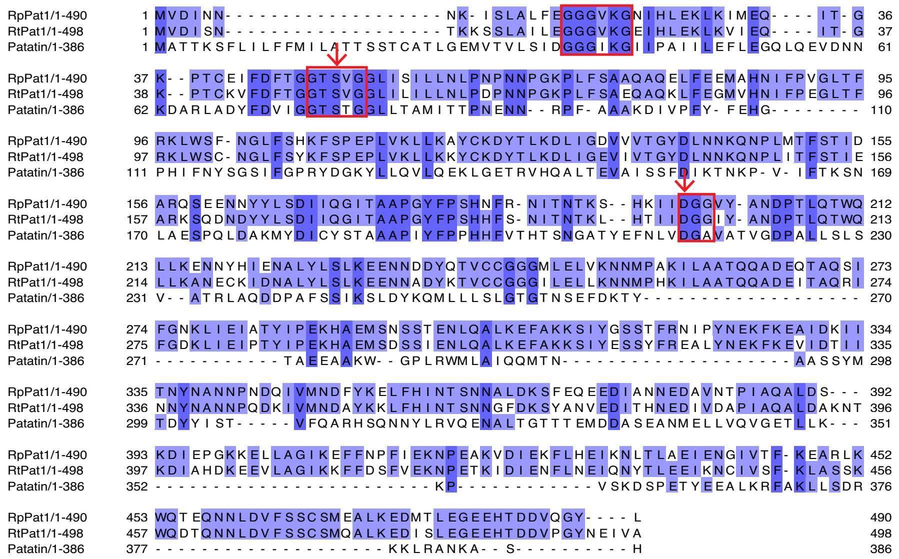

Supplementary Fig. 1 *R. parkeri* Pat1 is a PLA_2 enzyme. **(**a) Amino acid sequence alignment of Pat1 from *R. parkeri* strain Portsmouth (accession # WP_014410903.1), Pat1 from *R. typhi* strain Wilmington (accession # WP_011191036), and patatin from *Solanum tuberosum* (accession # P07745). Alignments were performed using CLUSTALW. Shading indicates amino acid sequence identity between two or three proteins at each position. Red boxes indicate (in order): glycine-rich motif (G-G-G-X-X-G) and serine hydrolase motif (G-X-S-X-G). Arrows indicate catalytic serine (S50 in *R. parkeri* Pat1) and aspartate (D198 in *R. parkeri* Pat1).

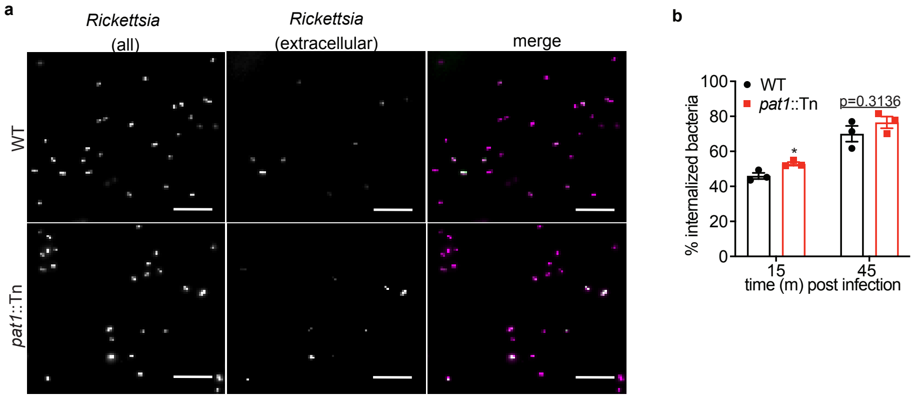

Supplementary Fig. 2 Pat1 is not important for invasion of host cells. (a) Images of intracellular/ extracellular staining to quantify invasion. Scale Bar is 10 μm. (b) Quantification of (a), percentage of bacteria internalized at 15 mpi and 45 mpi (>500 bacteria counted per strain/timepoint), MOI 5. Data are mean ± SEM (n=3 independent experiments); *p=0.0281 relative to WT (unpaired t-test (two-tailed)). Source data are provided as a Source Data file.

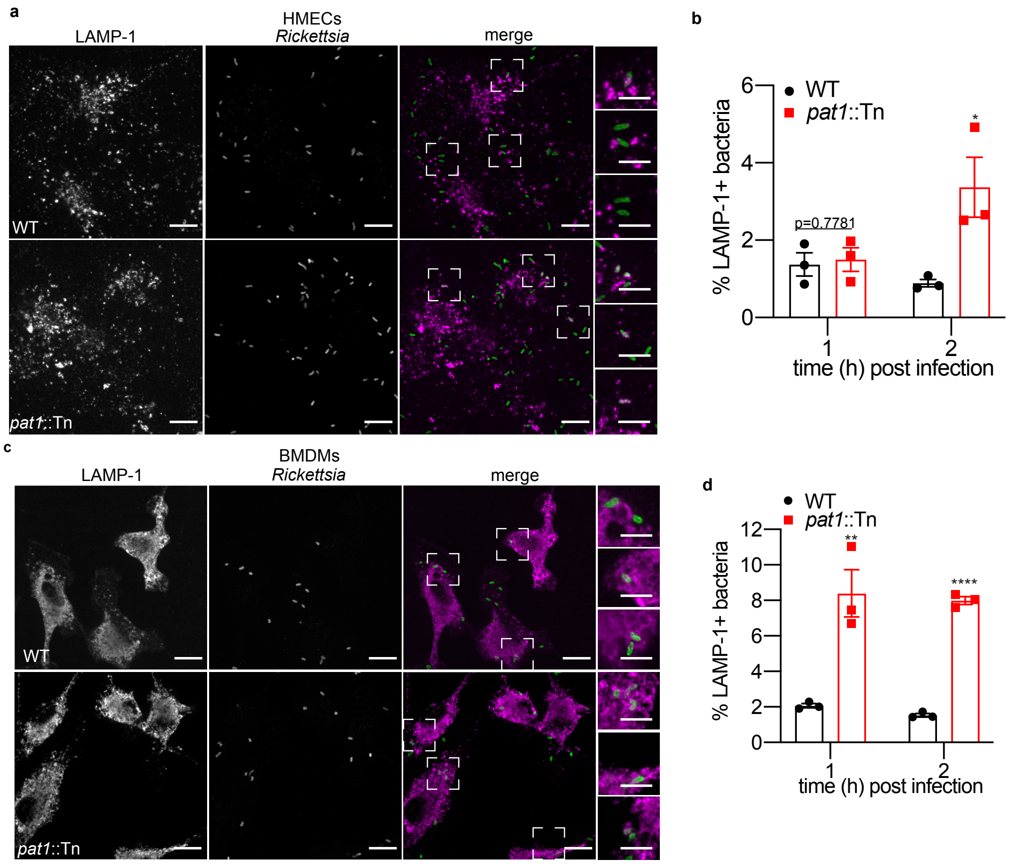

Supplementary Fig. 3 Pat1 enables avoidance of trafficking to late endosomal and lysosomal compartments. (a) Images of LAMP-1 (magenta in merge) in HMECs infected with WT or *pat1*::Tn bacteria (green in merge) at 2 hpi. Boxes indicate insets on right. (b) Quantification of LAMP-1 positive bacteria at 1 hpi (images not shown) and 2 hpi (images in (a); >1000 bacteria counted per strain/timepoint), MOI 5. (c) Images of LAMP-1 (magenta in merge) in BMDMs infected with WT or *pat1*::Tn bacteria (green in merge) at 2 hpi. Boxes indicate insets on right. (d) Quantification of LAMP-1 positive bacteria at 1 hpi (images not shown) and 2 hpi (images in (c); >1000 bacteria counted per strain/timepoint), MOI 5. Scale bar in (a) and (c) is 10 μm, inset 3 μm. All data represent n=3 independent experiments. Data in (b) and (d) are mean ± SEM; ****$p<0.0001$ **$p=0.0092$ *$p=0.0342$ relative to WT (unpaired t-test (two-tailed)). Source data are provided as a Source Data file.

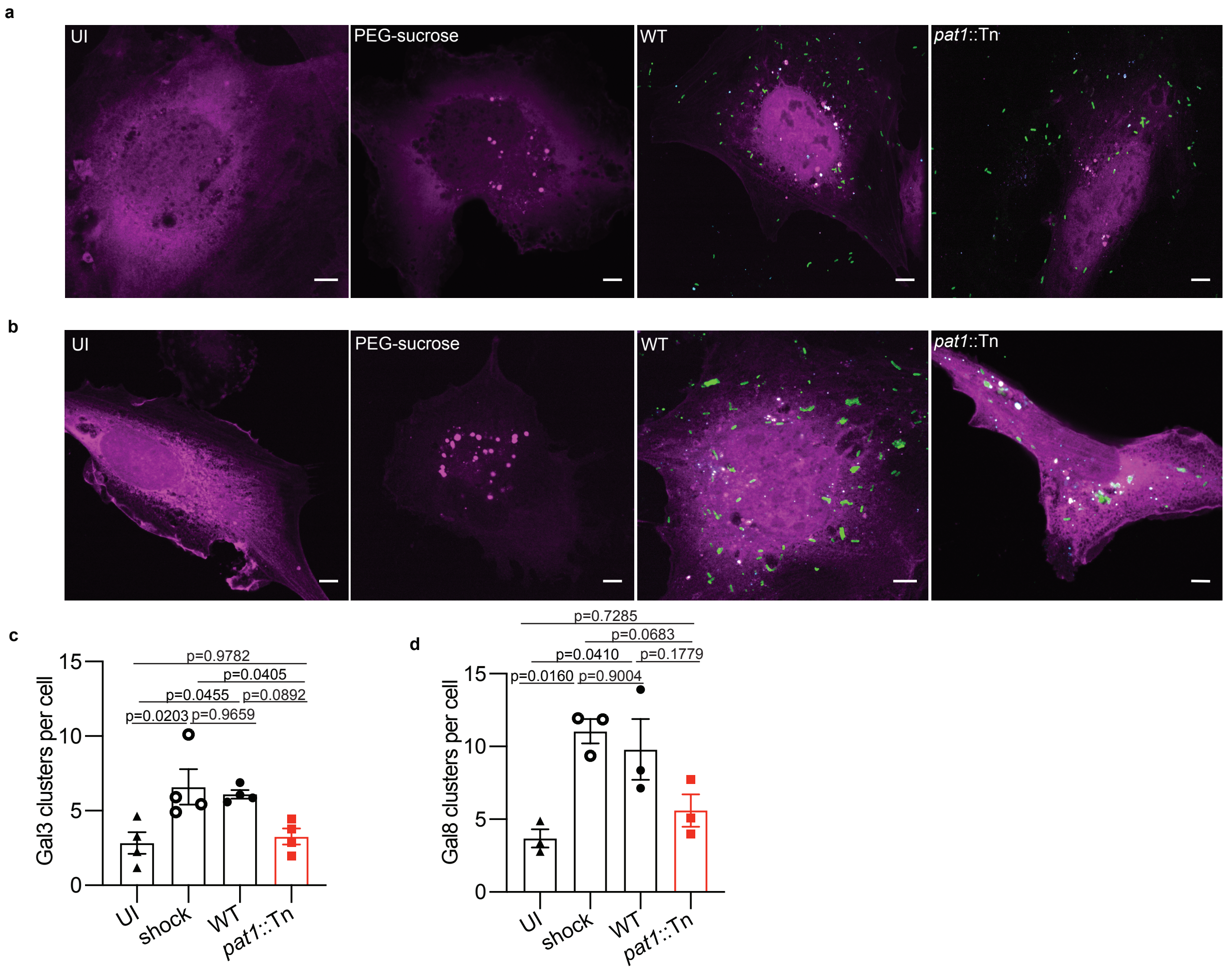

Supplementary Fig. 4 Pat1 contributes to intracellular membrane damage. Images of (a) Gal3-mCherry (magenta) and (b) Gal8-mCherry (magenta) in HMECs that are uninfected (UI), undergo sterile lysis of vesicles (hypotonic shock; PEG-sucrose), WT-infected, and *pat1*::Tn mutant-infected (green) infected at 1 hpi. Infected panels are also stained for NDP52 (cyan). Scale bars for (a) and (b) are 4 μm. (c) Number of Gal3 clusters per cell (n=4 independent experiments; >40 individual fields quantified). (d) Number of Gal8 clusters per cell (n=3 independent experiments; >30 individual fields quantified). Data in (c) and (d) are mean ± SEM (one-way ANOVA, multiple comparisons with Tukey post hoc test). Source data are provided as a Source Data file.

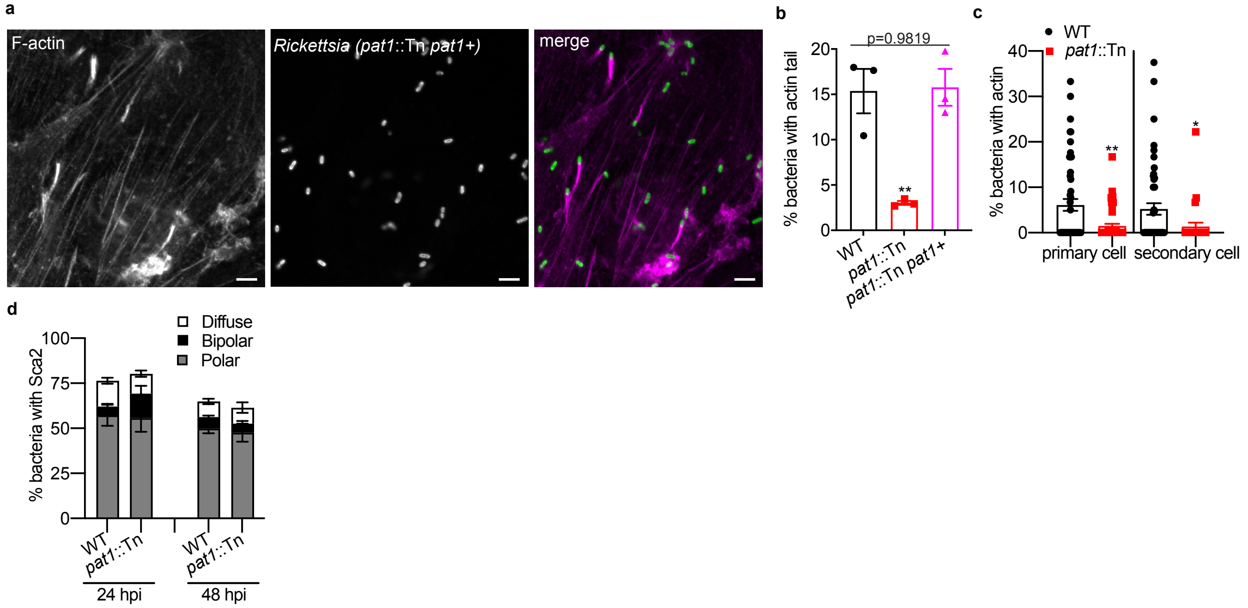

Supplementary Fig. 5 The frequency of actin-based motility for the *pat1*::Tn mutant is reduced in the primary and secondary cell and this is not due to altered localization of Sca2. (a) Images of actin tails in the complemented mutant (F-actin, magenta in merge; bacteria, green in merge) in HMECs at 48 hpi. (WT and *pat1*::Tn images represented in figure 6f) Scale bar is 5 µm. (b) Percentage of bacteria with actin tails at for the indicated strains (>1000 bacteria counted per strain), MOI 0.5. Data are mean ± SEM; **p=0.0059 relative to WT (one-way ANOVA with Dunnett's post hoc test). (c) Percentage of bacteria with actin tails in primary and secondary cells, related to Figure 6c, MOI 4. Data in (c) was quantified from individual image fields that contained bacteria in primary (left) and secondary cells (right) (primary cell n=51 fields for WT and 54 fields for *pat1*::Tn; secondary cell n= 46 fields for WT, n= 27 fields for *pat1*::Tn; bacterial number is in Figure 6e). Data are mean ± SEM; **p=0.0078 *p=0.0189 relative to WT (Mann-Whitney (two-tailed)). (d) Percentage of bacteria with Sca2 with the indicated distributions in WT and *pat1*::Tn mutant bacteria (images not shown; >200 bacteria counted per strain/timepoint),

MOI 0.5. Data are mean ± SEM. All data represent n=3 independent experiments. Source data are provided as a Source Data file.

Scanned unmodified images of blots used in Figure 1c

Anti-Pat1

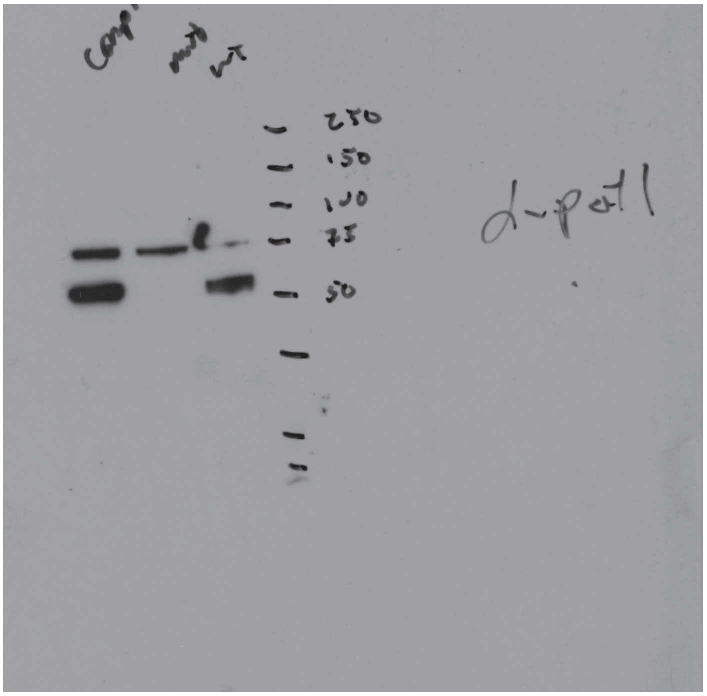

Anti-RickA

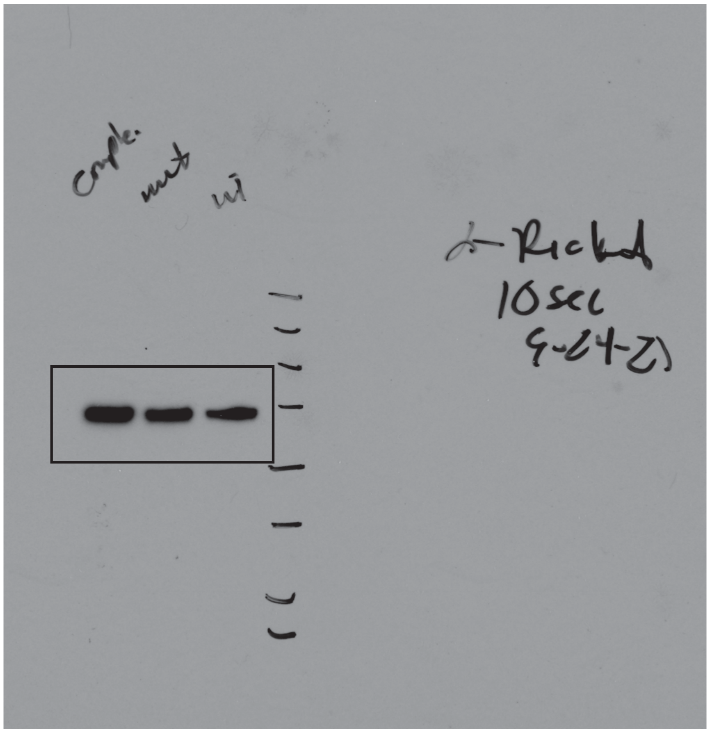

Supplement: Supplementary file 1 — Supplementary Information [file 41467_2022_31351_MOESM1_ESM.pdf]
