# Supplementary material for: A patatin-like phospholipase mediates Rickettsia parkeri escape from host membranes
Source: Nat Commun. 2022 Jun 27;13:3656. doi: 10.1038/s41467-022-31351-y (PMC9237051; doi:10.1038/s41467-022-31351-y)
Supplement: Supplementary file 2 — Reporting Summary [file 41467_2022_31351_MOESM2_ESM.pdf]

nature portfolio

Corresponding author(s): Matthew D. Welch
Last updated by author(s): May 30, 2022

Reporting Summary

Nature Portfolio wishes to improve the reproducibility of the work that we publish. This form provides structure for consistency and transparency in reporting. For further information on Nature Portfolio policies, see our Editorial Policies and the Editorial Policy Checklist.

Statistics

For all statistical analyses, confirm that the following items are present in the figure legend, table legend, main text, or Methods section.

n/a	Confirmed	
☐	☒	The exact sample size (*n*) for each experimental group/condition, given as a discrete number and unit of measurement
☐	☒	A statement on whether measurements were taken from distinct samples or whether the same sample was measured repeatedly
☐	☒	The statistical test(s) used AND whether they are one- or two-sided<br>*Only common tests should be described solely by name; describe more complex techniques in the Methods section.*
☒	☐	A description of all covariates tested
☐	☒	A description of any assumptions or corrections, such as tests of normality and adjustment for multiple comparisons
☐	☒	A full description of the statistical parameters including central tendency (e.g. means) or other basic estimates (e.g. regression coefficient) AND variation (e.g. standard deviation) or associated estimates of uncertainty (e.g. confidence intervals)
☐	☒	For null hypothesis testing, the test statistic (e.g. *F*, *t*, *r*) with confidence intervals, effect sizes, degrees of freedom and *P* value noted<br>*Give P values as exact values whenever suitable.*
☒	☐	For Bayesian analysis, information on the choice of priors and Markov chain Monte Carlo settings
☒	☐	For hierarchical and complex designs, identification of the appropriate level for tests and full reporting of outcomes
☒	☐	Estimates of effect sizes (e.g. Cohen's *d*, Pearson's *r*), indicating how they were calculated

Our web collection on statistics for biologists contains articles on many of the points above.

Software and code

Policy information about availability of computer code

Data collection	MetaMorph software version 7.8.2.0 (Molecular Devices)
Data analysis	PRISM 9 software version 9.3.1 (GraphPad Software, La Jolla, CA); ImageJ Version 2.1.0/153c; Adobe Illustrator version 26.3.1

For manuscripts utilizing custom algorithms or software that are central to the research but not yet described in published literature, software must be made available to editors and reviewers. We strongly encourage code deposition in a community repository (e.g. GitHub). See the Nature Portfolio guidelines for submitting code & software for further information.

Data

Policy information about availability of data

All manuscripts must include a data availability statement. This statement should provide the following information, where applicable:

- Accession codes, unique identifiers, or web links for publicly available datasets
- A description of any restrictions on data availability
- For clinical datasets or third party data, please ensure that the statement adheres to our policy

Source data are provided with this paper. The sequencing reads from whole genome sequencing of the bacterial strain pat1::Tn is publicly available at the Sequence Read Archive (SRA) as accession number SRR18465981 (https://www.ncbi.nlm.nih.gov/sra/?term=SRR18465981). For bacterial promoter prediction, SOFTBERRY BPROM was used (http://www.softberry.com/berry.phtml?topic=bprom&group=programs&subgr oup=gfindb). Bacterial terminator prediction was determined using the WebGeSTer datebase (http://pallab.serc.iisc.ernet.in/gester).

Field-specific reporting

Please select the one below that is the best fit for your research. If you are not sure, read the appropriate sections before making your selection.

- [x] Life sciences
- [ ] Behavioural & social sciences
- [ ] Ecological, evolutionary & environmental sciences

For a reference copy of the document with all sections, see nature.com/documents/nr-reporting-summary-flat.pdf

Life sciences study design

All studies must disclose on these points even when the disclosure is negative.

Sample size	No statistical methods were used to determine sample size; sample size determination was based on sample sizes used previously in similar experiments in the following publications (Lamason et al, Cell doi: 10.1016/j.cell.2016.09.023 (2016) and Engström et al, Nature. doi: 10.1038/s41564-019-0583-6 (2019)).
Data exclusions	There was no data exclusion in this study.
Replication	Each experiment was replicated at least two independent times (as indicated in figure legends). Replicated experiments were done on different days with different bacterial preparations.
Randomization	Randomization was used for mice experiments to randomly assign mice to infection with WT or mutant bacteria. For imaging experiments, imaged fields for quantification were randomly selected. Randomization was not relevant to biochemical or cell-based experiments and all bacterial strains were grown under the same conditions.
Blinding	Blinding was not used in any experiments presented in this paper since there were no patients or clinical trials.

Reporting for specific materials, systems and methods

We require information from authors about some types of materials, experimental systems and methods used in many studies. Here, indicate whether each material, system or method listed is relevant to your study. If you are not sure if a list item applies to your research, read the appropriate section before selecting a response.

Materials & experimental systems

n/a	Involved in the study	
☐	☒	Antibodies
☐	☒	Eukaryotic cell lines
☒	☐	Palaeontology and archaeology
☐	☒	Animals and other organisms
☒	☐	Human research participants
☒	☐	Clinical data
☒	☐	Dual use research of concern

Methods

n/a	Involved in the study	
☒	☐	ChIP-seq
☒	☐	Flow cytometry
☒	☐	MRI-based neuroimaging

Antibodies

Antibodies used	The following antibody was generated and affinity purified for this study: anti-Rickettsia Pat1, Pocono Rabbit Farm and Laboratory (Canadensis, PA) performed immunization and bleeds of rabbits immunized with purified Pat1. Antibodies previously generated in the lab and published: anti-Rickettsia OmpB, anti-Rickettsia RickA. Antibodies provided by other researchers: anti-Rickettsia I7205, anti-Rickettsia 14-13 (from Ted Hackstadt, NIH Rocky Mountain labs). Antibodies from commercial vendors: mouse anti-polyubiquitin FK1 (EMD Millipore, catalog number 04-262), guinea pig anti-p62 (Fitzgerald, catalog number 20RPP001), mouse anti-NDP52 (Novus Biologicals, catalog number H00010241-B01P), rabbit anti-LC3 (Novus Biologicals, catalog number NB100-2220SS), mouse anti-human LAMP-1 (BD Bioscience, catalog number 555801), mouse anti-β-catenin (BD Bioscience; catalog number 610153), mouse anti-LAMP-1 (Biolegend, catalog number 121609), rabbit anti-Calnexin (Enzo Life Sciences, catalog number ADI-SPA860-D), rabbit anti-protein disulfide isomerase (PDI)(Enzo Life Sciences, catalog number ADI-SPA-890-D). Goat anti-rabbit IgG-HRP (Santa Cruz Biotechnology, catalog number sc-2004) was used for western blotting on bacterial cell lysates.
Validation	The Pat1 antibody used in this study was validated by western blot (Fig. 1B). The OmpB antibody was previously validated for western blot and immunofluorescence in Engström et al, Nature. doi: 10.1038/s41564-019-0583-6 (2019). The RickA antibody was previously validated for western blot in Reed et al, Curr. Biol. doi:10.1016/j.cub.2013.11.025 (2014). All of the following antibodies were previously used for immunofluorescence in Engström et al, Nature. doi: 10.1038/s41564-019-0583-6. (2019) and further validation details are given for each antibody below. The polyubiquitin FK1 antibody had been validated for western blot experiments and immunofluorescence microscopy experiments, as indicated by the manufacturer's website. The p62 antibody was previously used in immunofluorescence microscopy experiments in Mitchell et al, Proc Natl Acad Sci U S A. doi: 10.1073/pnas.1716055115 (2018); and Cheng et al, Cell Microbiol. doi: 10.1111/cmi.12854 (2018). The NDP52 antibody had been validated for western blot experiments by the manufacturer and were used for immunofluorescence microscopy in references Meunier et al, Nature. doi: 10.1038/

nature13157 (2014); and Manzanillo et al, Nature. doi: 10.1038/nature12566 (2013). The rabbit anti-LC3 antibody is LC3B knockout validated (for both western and IF) by the manufacturer. The mouse anti-Human CD107a (LAMP-1) antibody was quality tested by the manufacturer for flow cytometry and validated for immunofluorescence microscopic analysis in Kortebi et al, PLoS Pathog. doi: 10.1371/ journal.ppat.1006734 (2017). Anti-β-catenin was tested by the manufacturer for immunofluorescence during development and was used in microscopy experiments in the following publications: Lamason et al, Cell doi: 10.1016/j.cell.2016.09.023 (2016) and Eger at al, J Cell Bio. doi: 10.1083/jcb.148.1.173 (2000). Anti-mouse LAMP-1 was quality tested by manufacturer for immunofluoresence staining with flow cytometry and was used for immunofluorescence microscopic analysis in references Mitchell et al, Infection and Immunity. doi: 10.1128/IAI.00110-15 (2015) and Engström et al, Nature. doi: 10.1038/s41564-019-0583-6 (2019). Anti-Calnexin and anti-PDI was quality tested by manufacturer for multiple applications and used for immunofluorescence staining in cells in reference Meunier and Broz, J Vis Exp. doi: 10.3791/52960 (2015). Goat anti-rabbit IgG-HRP was tested by manufacturer.

Eukaryotic cell lines

Policy information about cell lines

Cell line source(s)	HMEC-1, A549, and Vero cell lines were purchased from UC Berkeley Cell Culture Facility (https://bds.berkeley.edu/facilities/cell-culture). BMDM were isolated from the femurs of female C57BL/6 mice (Charles River Laboratories, Wilmington, MA).
Authentication	Authentication by short tandem repeat analysis was performed using Promega GenePrint system (authentication done by the UC Berkeley Cell Culture facility).
Mycoplasma contamination	Cells were inspected and confirmed negative for Mycoplasma contamination using DAPI stain and fluorescent microscopy by the cell culture facility.
Commonly misidentified lines (See ICLAC register)	No commonly misidentified cell lines were used in this study.

Animals and other organisms

Policy information about studies involving animals; ARRIVE guidelines recommended for reporting animal research

Laboratory animals	C57BL/6 background and carried mutations in the genes encoding the receptors for IFN-I (Ifnar) and IFN-γ (Ifngr) (Ifnar-/-Ifngr-/-) (described in Burke et al Nature Micro. doi: 10.1038/s41564-020-0673-5 (2020). Mice were between 8 and 20 weeks old at the time of initial infection and no mouse was excluded due to sex and experiments had as close to 50% male/female as possible.
Wild animals	No wild animals were used in this study.
Field-collected samples	No field-collected samples were used in this study.
Ethics oversight	Animal work was approved and monitored by University of California, Berkeley Institutional Animal Care and Use Committee (IACUC) in compliance with the Animal Welfare Act and other federal statutes relating to animals and experiments using animals (Welch lab animal use protocol AUP-2016-02-8426).

Note that full information on the approval of the study protocol must also be provided in the manuscript.
